# Supplementary material for: Activating de novo mutations in NFE2L2 encoding NRF2 cause a multisystem disorder
Source: Nat Commun. 2017 Oct 10;8:818. doi: 10.1038/s41467-017-00932-7 (PMC5635015; doi:10.1038/s41467-017-00932-7)
Supplement: Supplementary file 1 — Supplementary Information [file 41467_2017_932_MOESM1_ESM.pdf]

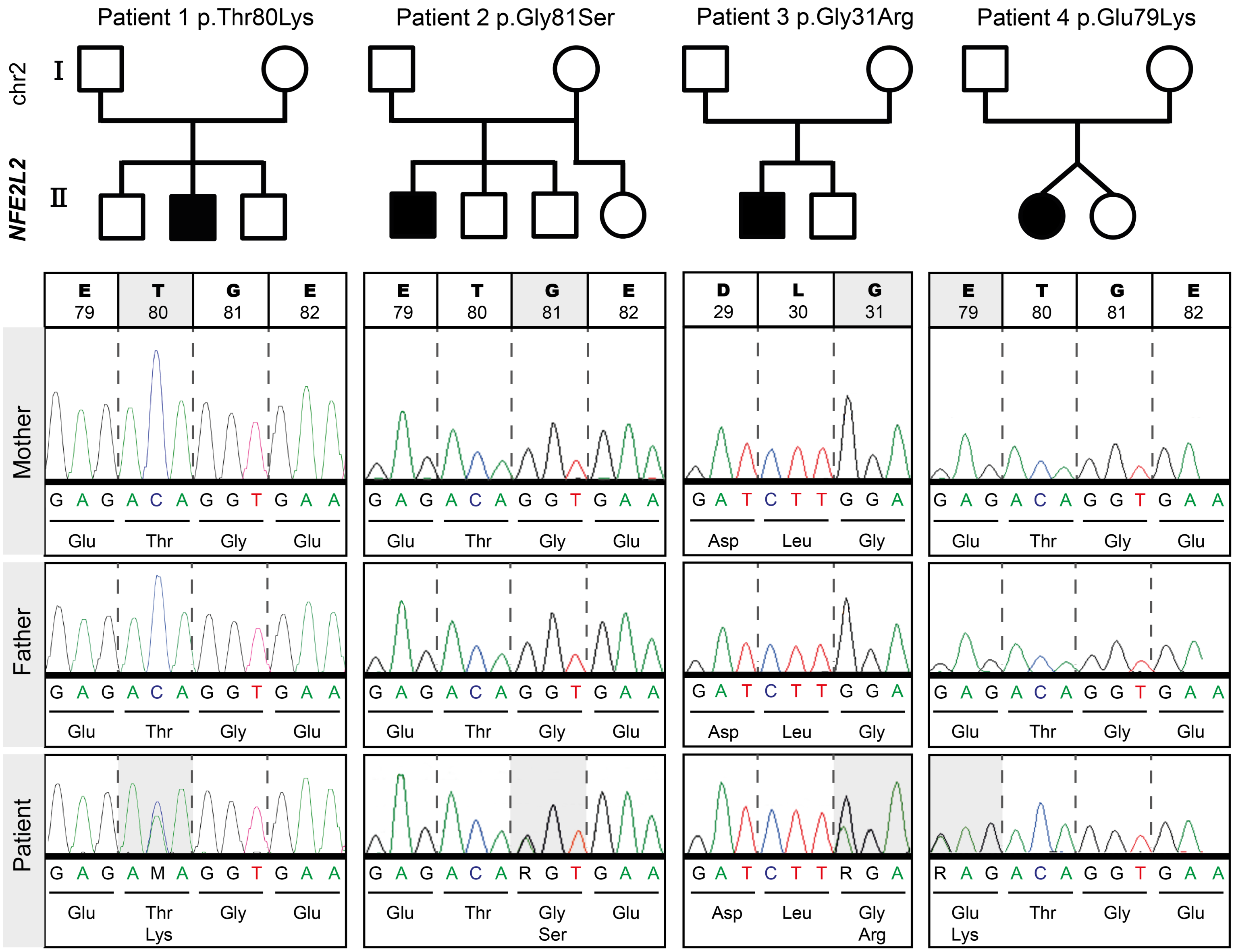

Supplementary Figure 1 Pedigrees and confirmation of *NFE2L2* variants. Pedigrees of the four *NFE2L2* mutant patients and their families with the corresponding Sanger sequencing chromatogram of the mutant DLG/ETGE motif of the patient-parent Trio below. The position of the affected nucleotide is highlighted by a grey box. Nomenclature is according to GenBank accession number NM_006164.4 and NP_006155.2.

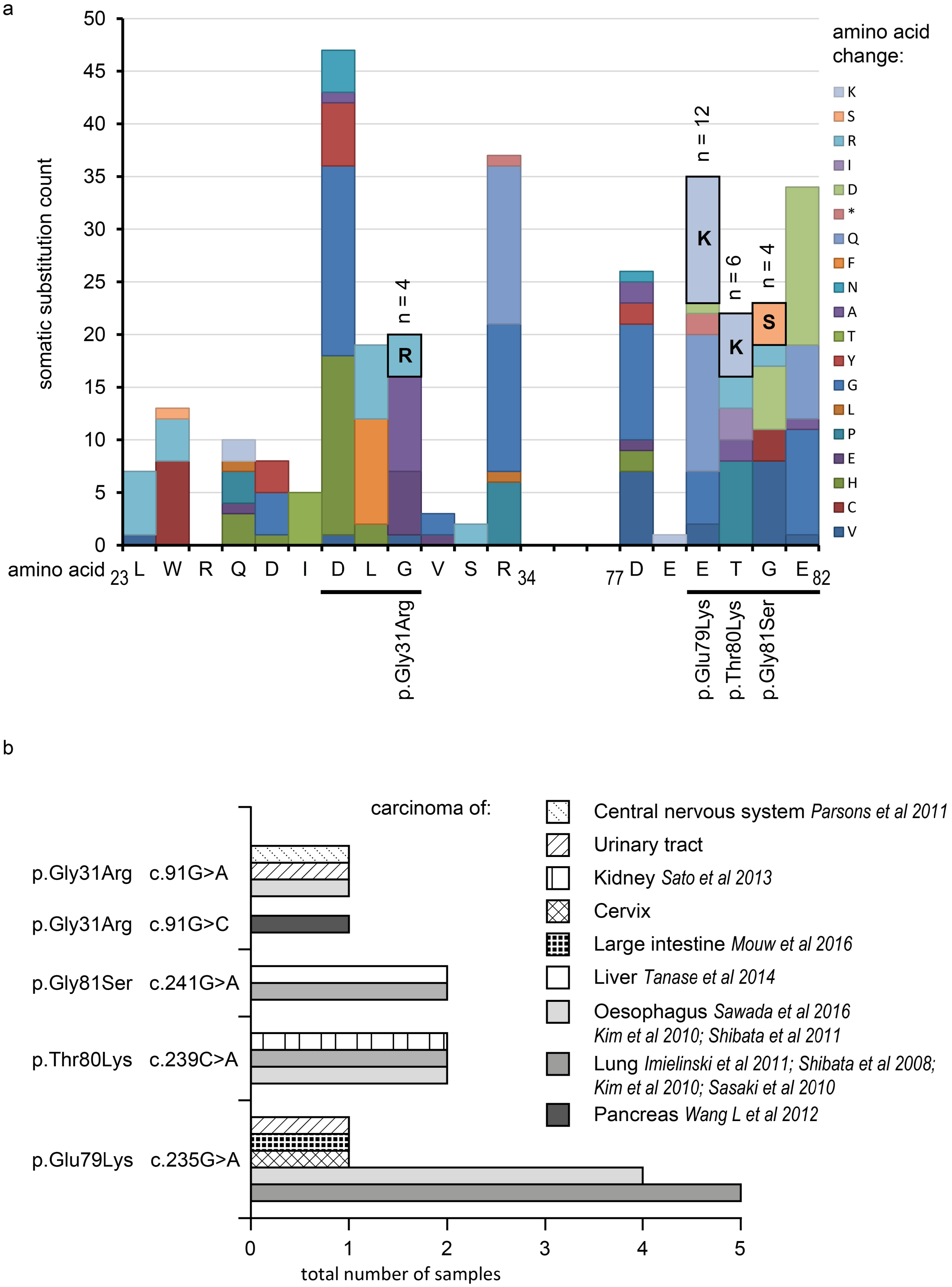

Supplementary Figure 2 Somatic cancer associated NRF2 substitutions in the DLG and ETGE motif. (**a**) Overview of cancer related somatic substitutions in the DLG and ETGE motif of NRF2. The variants that resemble the pathogenic mutations described in this article are highlighted by a white box. The number indicates how often the mutations have been described in cancer cells. (**b**) Showing in which kind of cancer and how frequently the mutations described in this article have been reported previously. Data was summarized using the COSMIC database.

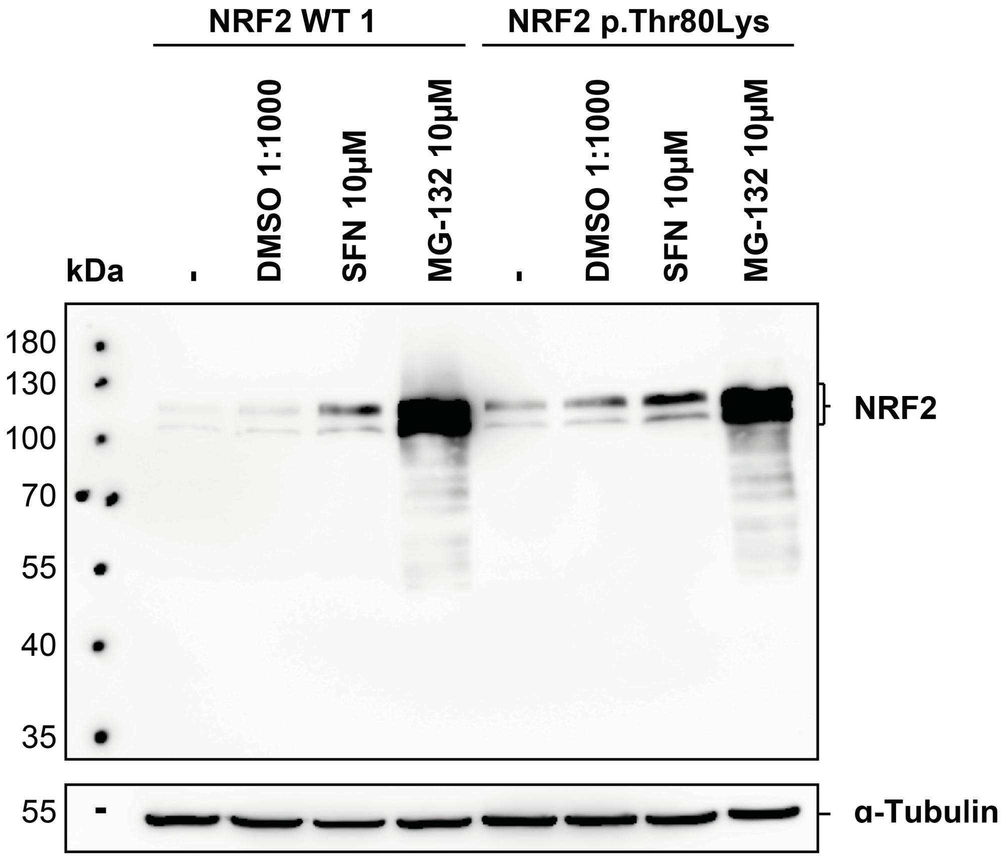

Supplementary Figure 3 Validation of NRF2 antibody. NRF2 p.T80K mutant and wild type primary fibroblast cell lines were treated with SFN (10µM) and MG-132 (10µM) for 16h. As negative control DMSO treated and untreated cells were used. 25µg of whole protein lysates were immunoblotted and increased signals for NRF2 at ~100kDa for SFN and MG-132 treated cells were determined. αTubulin was used as loading control.

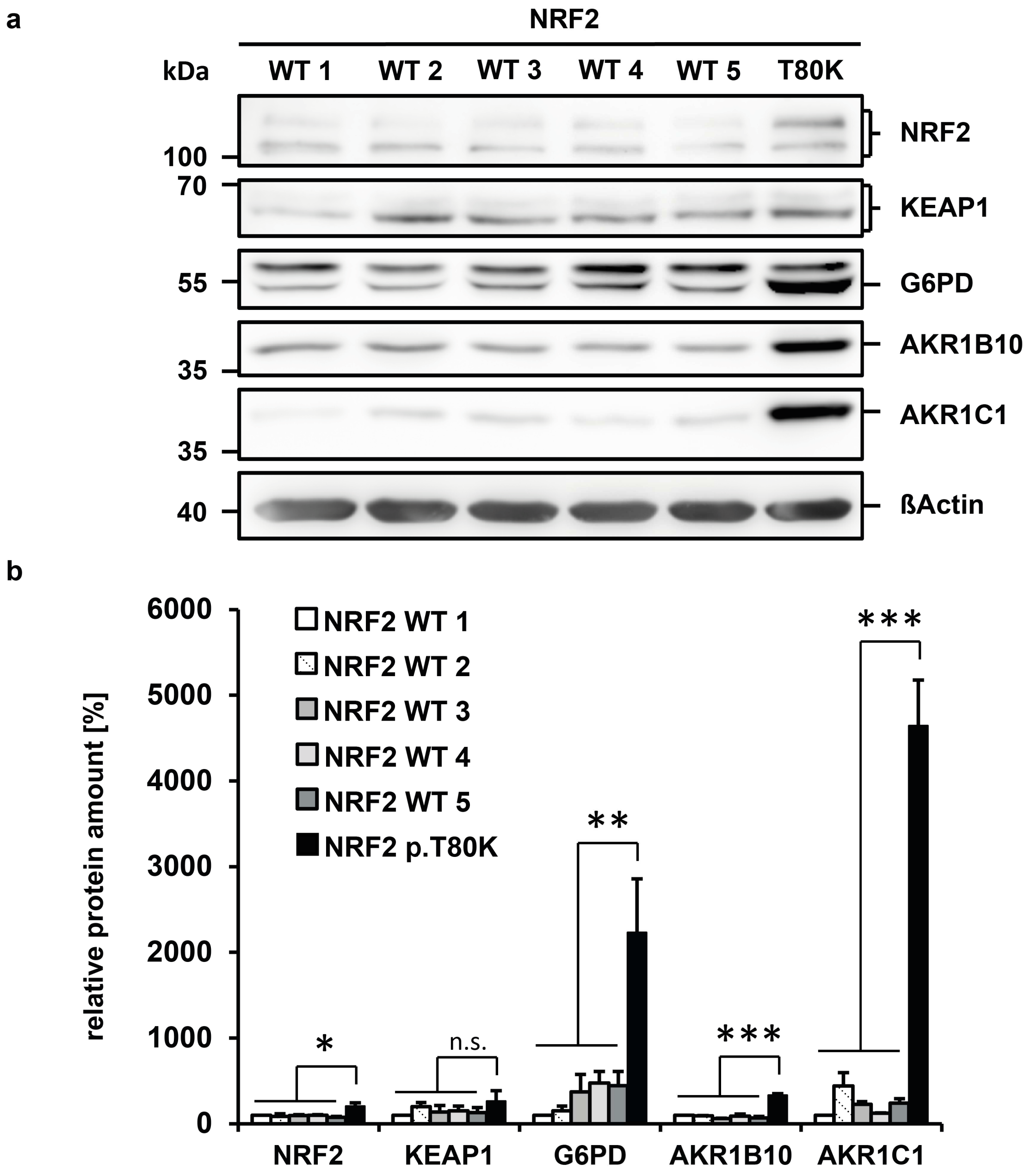

Supplementary Figure 4 Increased stabilization of mutant NRF2. (**a**) Representative Western blot of endogenous level of NRF2, KEAP1, G6PD, AKR1B10 and AKR1C1 in 25µg whole protein lysates of human primary fibroblast cell lines from five controls (NRF2 WT 1 - WT 5) and patient 1 with NRF2 p.T80K variant. (**b**) Quantitative analysis of western blot images illustrating the endogenous level of NRF2, KEAP1, G6PD, AKR1B10 and AKR1C1 relative normalized to ACTB and NRF2 WT 1. Data are given as means ± SEM, n = 3 independent experiments. Data were analyzed by one-way ANOVA with multiple comparisons: $p \leq 0.05$ *, $p \leq 0.01$ **, $p \leq 0.001$ ***.

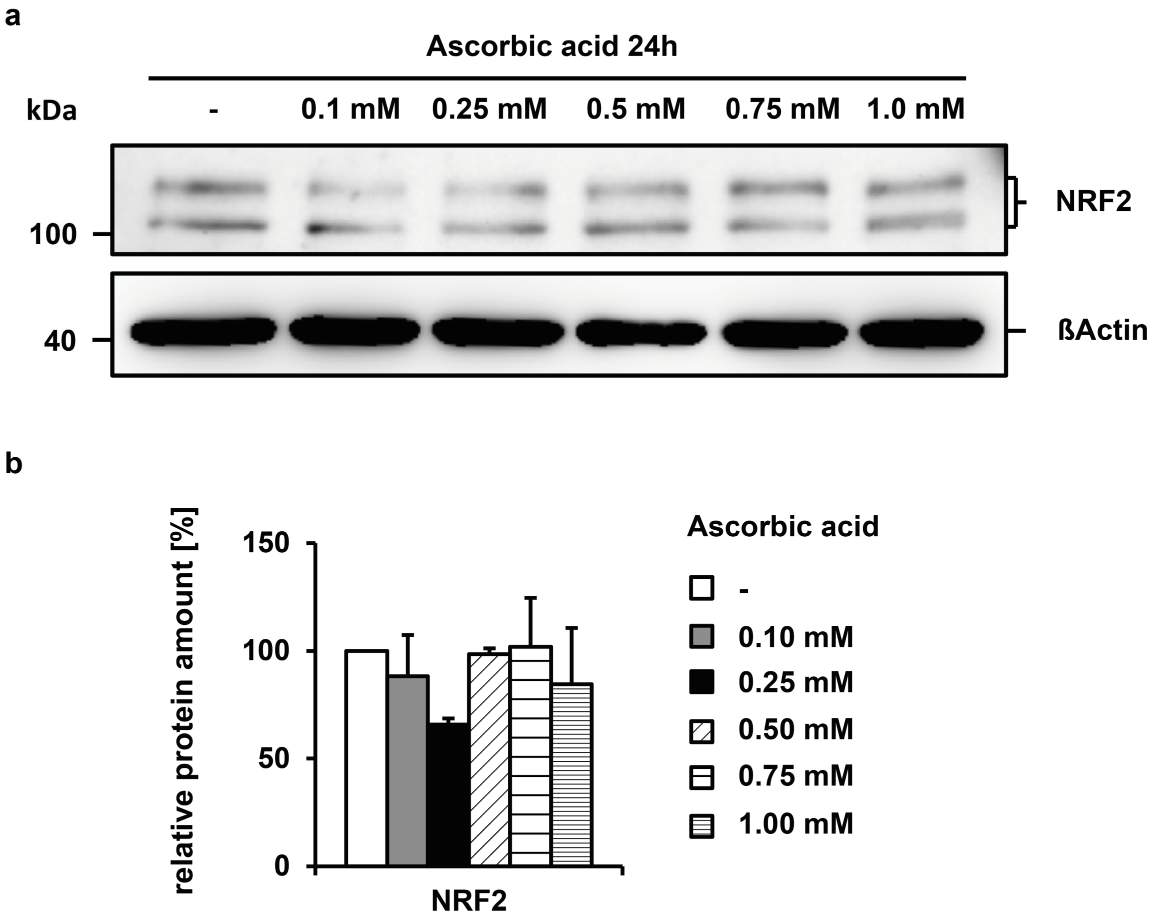

Supplementary Figure 5 Effect of ascorbic acid on NRF2 level (**a**). Representative Western blot of endogenous level of NRF2 in 15µg whole protein lysates of treated human primary fibroblast of patient 1. NRF2 p.T80K mutant cells were exposed to 0.1mM, 0.25mM, 0.5mM, 0.75mM, 1mM ascorbic acid or without for 24h. (**b**) Quantitative analysis of western blot images illustrating the endogenous level of NRF2 relative normalized to ACTB and untreated cells. Data are given as means ± SEM, n = 3 independent experiments. Data were analyzed by one-way ANOVA with multiple comparisons: $p \leq 0.05$ *, $p \leq 0.01$ **, $p \leq 0.001$ ***.

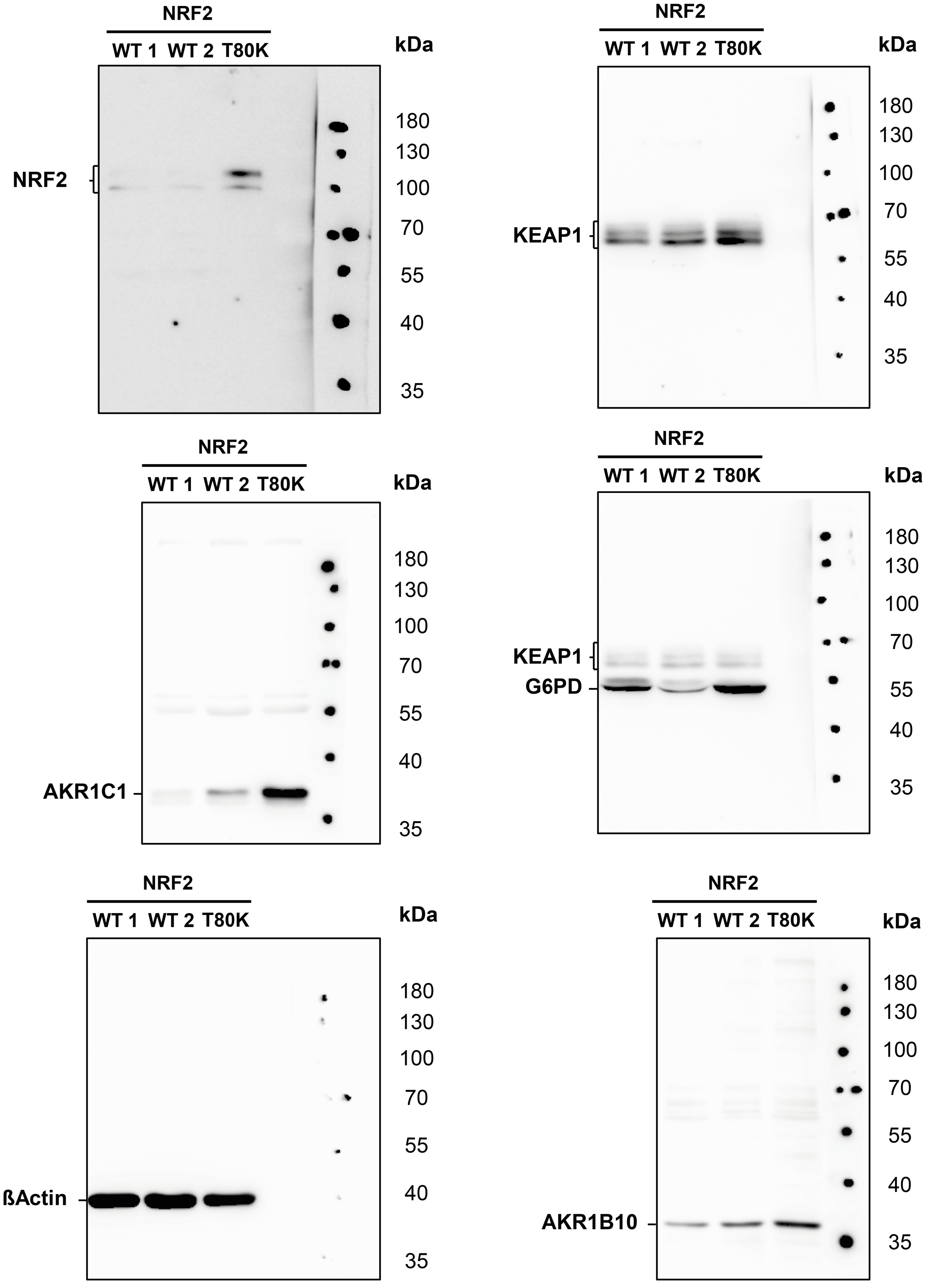

Supplementary Figure 6 Full blots of protein expression of NRF2, KEAP1, G6PD, AKR1B10 and AKR1C1 in human primary fibroblast cell lines from two controls (NRF2 WT 1, WT 2) and patient 1 with NRF2 p.T80K variant.

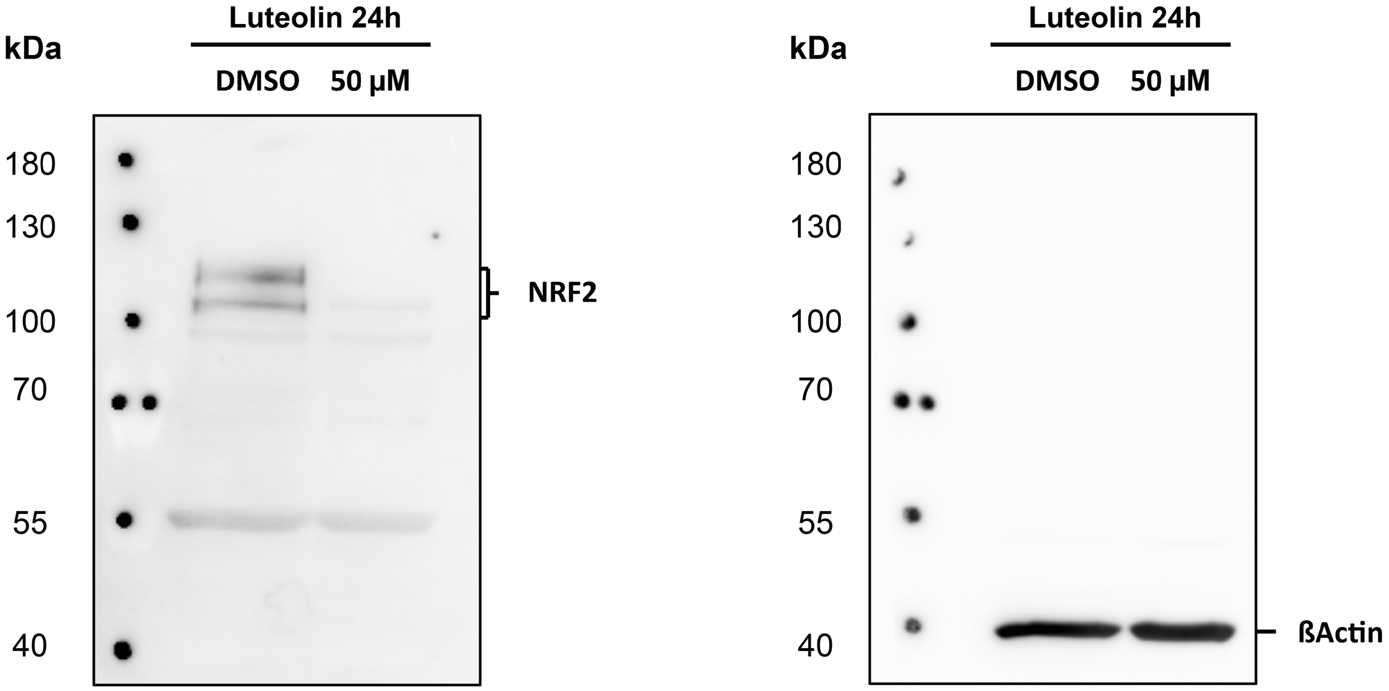

Supplementary Figure 7 Full blots of protein expression of NRF2 of treated human primary fibroblast of patient 1. NRF2 p.T80K mutant cells were exposed to 50µM luteolin or DMSO for 24h.

Sample	Cov 30X	Mean Cov	Total reads	Unique mapped reads	Autosomal Runs of Homozygosity [Mb]	% of rare hom Variants	Gender
Patient	81.3	72	15100972	11915001	69	1.4	Male
Mother	80.3	71	14444790	11441169	77	1.2	Female
Father	86.8	80	16013178	12346597	160	2.8	Male

Supplementary Table 1 Basic Mendeliome sequencing output data. Overview of coverage, mapping information and homozygosity for each mendeliome sample. Consanguinity can be estimated using % of rare hom variants (3 – 6%) and autosomal runs of homozygosity (> 200 – 300 mb). Cov, coverage; hom, homozygosity

	Gene	Chromosome	Position (hg19)	Reference allele	Mutant allele	Transcript ID	Variant	Mutation status		
								Patient	Mother	Father
de novo	***PIGR***	1	207105819	G	A	NM_002644.3	p.R664W	het	WT	WT
	NFE2L2	2	178098806	G	T	NM_006164.4	p.T80K	het	WT	WT
	CCDC170	6	151869623	A	G	NM_025059.3	p.Q258R	het	WT	WT
hemizygous	***ARSE***	X	2856166	C	T	NM_000047.2	p.R420Q	hom	het	WT
	CYBB	X	37664243	C	T	NM_000397.3	c.1152-16C>T	hom	het	WT
	ZNF674	X	46360758	A	G	NM_001146291.1	p.V83A	hom	het	WT
	CHRDL1	X	110035391	T	C	NM_145234.3	p.M7V	hom	het	WT
compound heterozygous	***NCAPD2***	12	6618863	T	A	NM_014865.3	c.128-20T>A	het	het	WT
	NCAPD2	12	6638102	G	C	NM_014865.3	c.3478-9G>C	het	WT	het
	RELN	7	103155698	A	G	NM_005045.3	p.S2685P	het	het	WT
	RELN	7	103153833	C	A	NM_005045.3	c.7490+5G>T	het	WT	WT

Supplementary Table 2a

Supplementary Table 2 Detected filtered *de novo*, compound heterozygous and homozygous variants. (**a**) Detailed variant information with localization, substitution and occurrence within the family as well as (**b**) corresponding bioinformatic prediction on the functional effect, conservation and occurrence in ExAC. Variant classification for each tool was obtained using dbNSFP version 3.0a. Het, heterozygous; hom, homozygous; WT, wild type; B, benign; N, neutral; T, tolerated; D, damaging; H, high functional impact; n.a., not available

	Gene	Transcript ID	Variant	GERP>2	SIFT_pred	Polyphen2_pred	LRT_pred	MutationTaster_pred	MutationAssessor_pred	FATHMM_pred	PROVEAN_pred	fathmm-MKL_coding_pred	MetaSVM_pred	MetaLR_pred	ExAC_AF
de novo	***PIGR***	NM_002644.3	p.R664W	+	D	D	D	D	M	T	D	D	T	T	0.000008
	NFE2L2	NM_006164.4	p.T80K	+	D	D	D	D	M	T	D	D	T	T	-
	CCDC170	NM_025059.3	p.Q258R	+	T	B	D	D	L	T	N	D	T	T	-
hemizygous	***ARSE***	NM_000047.2	p.R420Q	-	T	B	N	N	N	D	N	N	T	T	0.000603
	CYBB	NM_000397.3	c.1152-16C>T	-					n.a.						-
	ZNF674	NM_001146291.1	p.V83A	-	T	B	.	N	L	T	N	N	T	T	0.000046
	CHRDL1	NM_145234.3	p.M7V	-	T	B	N	N	.	T	N	D	T	T	0.000433
compound heterozygous	***NCAPD2***	NM_014865.3	c.128-20T>A	-					n.a.						-
	NCAPD2	NM_014865.3	c.3478-9G>C	-					n.a.						-
	RELN	NM_005045.3	p.S2685P	+	D	B	N	D	N	T	N	D	T	T	0.000025
	RELN	NM_005045.3	c.7490+5G>T	+					n.a.						-

Supplementary Table 2b

			Mutation				
**Patient**	**Gene**	**Status**	**Chromosomal level**	**mRNA level**	**Protein level**	**Motif**	**Exon**
**Patient 1**	*NFE2L2*	*de novo*	chr2: 178098806 G>T	c.239C>A	p.T80K	ETGE	CDS.2
**Patient 2**	*NFE2L2*	*de novo*	chr2: 178098804 C>T	c.241G>A	p.G81S	ETGE	CDS.2
**Patient 3**	*NFE2L2*	*de novo*	chr2: 178098954 C>T	c.91G>A	p.G31R	DLG	CDS.2
**Patient 4**	*NFE2L2*	*de novo*	chr2: 178098810 C>T	c.235G>A	p.E79K	ETGE	CDS.2

Supplementary Table 3 Overview of detected pathogenic *NFE2L2* variants. Four detected heterozygous *NFE2L2 de novo* variants with detailed information regarding localization. Reference genome is hg19/ GRCh37. Nomenclature is according to GenBank accession number NM_006164.4 and NP_006155.2.

a Conservation Scores

Tool	Range	NRF2 Variant<br>Patient 1<br>p.T80K	 Patient 2<br>p.G81S	 Patient 3<br>p.G31R	 Patient 4<br>p.E79K	Prediction
PhastCons	0 - 1	0.999000	0.998000	1	1	**High conserved**
GERP	-12.3 – 6.17	5.78	5.78	5.78	5.78	**High conserved**

b Functional Prediction Algorithms

Tool	Range	NRF2 Variant<br>Patient 1<br>p.T80K	 Patient 2<br>p.G81S	 Patient 3<br>p.G31R	 Patient 4<br>p.E79K	Prediction
SIFT	0 - 1	0.00	0.00	0.00	0.00	**Damaging**
PROVEAN	-14 - 14	-3.99	-3.88	-5.53	-2.57	**Damaging**
PolyPhen2	0 - 1	1.00	1.00	1.00	1.00	**Probably damaging**
LRT	0 - 1	0.00	0.00	0.00	0.00	**Deleterious**
MutTaster	0 - 1	1.00	1.00	1.00	1.00	**Disease causing**
MutAssessor	-5.545 – 5.975	2.87	2.87	2.87	2.87	**Medium functional impact**
FATHMM	-16.13 – 10.64	1.43	1.22	1.37	1.48	**Tolerated**
VEST	0 - 1	0.81	0.79	0.94	0.88	**Damaging**
MetaSVM	-2 - 3	-0.52	-0.23	-0.42	-0.53	**Tolerated**
MetaLR	0 - 1	0.26	0.38	0.30	0.26	**Tolerated**

Supplementary Table 4 Bioinformatic prediction for identified NRF2 variants. Detected NRF2 variants were assessed regarding (**a**) conservation of affected residues and (**b**) functional effect of the variant. Scores of different software tools were obtained using dbNSFP version 3.0a. The software tools with the corresponding value range are listed. Mutation specific scores and the prediction model output for each tool are listed in the final row.

Figure 3

Figure 3b **Western blot**

Independent Experiment	Sample	NRF2 [%]	KEAP1 [%]	G6PD [%]	AKR1B10 [%]	AKR1C1 [%]
1	NRF2 WT 1	100.00	100.00		100.00	
	NRF2 WT 2	161.93	137.75		41.49	
	NRF2 p.T80K	1260.20	172.42		194.81	
2	NRF2 WT 1	100.00	100.00		100.00	
	NRF2 WT 2	118.21	204.37		61.12	
	NRF2 p.T80K	491.25	231.02		444.76	
3	NRF2 WT 1	100.00	100.00	100.00	100.00	100.00
	NRF2 WT 2	87.39	109.53	24.87	97.21	396.85
	NRF2 p.T80K	182.36	232.64	319.89	709.65	3098.74
4	NRF2 WT 1	100.00	100.00	100.00	100.00	100.00
	NRF2 WT 2	58.81	185.68	51.87	187.94	404.74
	NRF2 p.T80K	1128.84	168.00	226.38	496.33	2036.32
5	NRF2 WT 1	100.00	100.00	100.00	100.00	100.00
	NRF2 WT 2	104.85	141.84	81.87	75.60	516.64
	NRF2 p.T80K	248.98	271.38	427.98	115.77	3131.18
Mean	NRF2 WT 1	100.00	100.00	100.00	100.00	100.00
	NRF2 WT 2	106.24	155.84	52.87	92.67	439.41
	NRF2 p.T80K	662.33	215.09	324.75	392.26	2755.41
±SEM	NRF2 WT 1	0.00	0.00	0.00	0.00	0.00
	NRF2 WT 2	17.11	17.20	16.46	25.50	38.68
	NRF2 p.T80K	224.23	19.71	58.25	107.18	359.67
**One-Way Anova**	$p \le 0.05$ considered significant	$F(2,12)=6.184$, $p=0.014$	$F(2,12)=14.526$, $p=0.001$	$F(2,6)=17.284$, $p=0.003$	$F(2,12)=7.218$, $p=0.009$	$F(2,6)=47.878$, $p=0.000$
**multiple comparison post hoc test**	NRF2 p.T80K vs. WT 1	$p=0.025$	$p=0.000$	$p=0.009$	$p=0.018$	$p=0.000$
	NRF2 p.T80K vs. WT 2	$p=0.026$	$p=0.041$	$p=0.004$	$p=0.015$	$p=0.001$
	NRF2 WT 1 vs. WT 2	$p=0.999$	$p=0.055$	$p=0.630$	$p=0.996$	$p=0.522$
	$p \le 0.05$ *, $p \le 0.01$ **, $p \le 0.001$ ***	*	*	**	*	***

Figure 3c **qRT-PCR**

Independent Experiment	Sample	*GAPDH* [%]	*PGK1* [%]	*NFE2L2* [%]	*KEAP1* [%]	*GCLM* [%]	*GSR* [%]	*G6PD* [%]	*ME1* [%]	*PRDX1* [%]	*TXNRD1* [%]	*ABCC1* [%]	*HMOX1* [%]	*AKR1B10* [%]	*AKR1C1* [%]
1	NRF2 WT 1	100.00	100.00	100.00	100.00	100.00	100.00	100.00	100.00	100.00	100.00	100.00	100.00	100.00	100.00
	NRF2 WT 2	92.58	113.03	120.42	126.98	111.86	85.77	89.93	123.98	101.15	70.80	110.10	105.96	199.13	192.75
	NRF2 p.T80K	99.18	114.97	109.84	118.47	148.09	114.18	167.81	158.31	144.89	180.77	134.88	103.16	2534.69	763.35
2	NRF2 WT 1	100.00	100.00	100.00	100.00	100.00	100.00	100.00	100.00	100.00	100.00	100.00	100.00	100.00	100.00
	NRF2 WT 2	100.78	100.60	97.83	122.09	97.07	101.69	84.68	100.20	99.87	61.14	79.36	70.60	229.70	327.33
	NRF2 p.T80K	118.76	114.05	122.01	143.90	193.11	175.15	203.00	182.73	167.13	173.39	177.98	129.73	9162.11	2059.86
3	NRF2 WT 1	100.00	100.00	100.00	100.00	100.00	100.00	100.00	100.00	100.00	100.00	100.00	100.00	100.00	100.00
	NRF2 WT 2	86.13	91.35	104.46	138.08	85.43	126.15	106.41	106.52	101.57	61.29	110.63	76.25	207.20	387.89
	NRF2 p.T80K	123.57	118.94	183.45	213.09	226.91	227.15	300.63	270.64	218.84	147.69	283.62	236.09	5426.49	1660.21
Mean	NRF2 WT 1	100.00	100.00	100.00	100.00	100.00	100.00	100.00	100.00	100.00	100.00	100.00	100.00	100.00	100.00
	NRF2 WT 2	93.16	101.66	107.57	129.05	98.12	104.54	93.68	110.23	100.86	64.41	100.03	84.27	212.01	302.66
	NRF2 p.T80K	113.84	115.99	138.43	158.49	189.37	172.16	223.82	203.89	176.95	167.28	198.82	156.33	5707.76	1494.48
±SEM	NRF2 WT 1	0.00	0.00	0.00	0.00	0.00	0.00	0.00	0.00	0.00	0.00	0.00	0.00	0.00	0.00
	NRF2 WT 2	4.24	6.28	6.70	4.73	7.65	11.74	6.55	7.11	0.51	3.20	10.34	10.97	9.15	57.67
	NRF2 p.T80K	7.46	1.50	22.78	28.27	22.83	32.64	39.73	34.11	21.91	10.02	44.18	40.61	1918.33	383.33
**One-Way Anova**	$p \le 0.05$ considered significant	$F(2,6)=4.521$, $p=0.063$	$F(2,6)=5.558$, $p=0.043$	$F(2,6)=2.205$, $p=0.191$	$F(2,6)=3.122$, $p=0.118$	$F(2,6)=14.074$, $p=0.005$	$F(2,6)=4.071$, $p=0.076$	$F(2,6)=9.963$, $p=0.012$	$F(2,6)=8.101$, $p=0.020$	$F(2,6)=12.198$, $p=0.008$	$F(2,6)=73.953$, $p=0.000$	$F(2,6)=4.742$, $p=0.058$	$F(2,6)=2.433$, $p=0.168$	$F(2,6)=8.378$, $p=0.018$	$F(2,6)=11.333$, $p=0.009$
**multiple comparison post hoc test**	NRF2 p.T80K vs. WT 1	$p=0.199$	$p=0.052$	$p=0.197$	$p=0.102$	$p=0.009$	$p=0.096$	$p=0.022$	$p=0.025$	$p=0.012$	$p=0.001$	$p=0.083$	$p=0.301$	$p=0.027$	$p=0.011$
	NRF2 p.T80K vs. WT 2	$p=0.058$	$p=0.077$	$p=0.319$	$p=0.466$	$p=0.008$	$p=0.118$	$p=0.018$	$p=0.038$	$p=0.013$	$p=0.000$	$p=0.083$	$p=0.170$	$p=0.029$	$p=0.022$
	NRF2 WT 1 vs. WT 2	$p=0.617$	$p=0.947$	$p=0.920$	$p=0.474$	$p=0.995$	$p=0.986$	$p=0.980$	$p=0.932$	$p=0.999$	$p=0.014$	$p=1.000$	$p=0.893$	$p=0.997$	$p=0.804$
	$p \le 0.05$ *, $p \le 0.01$ **, $p \le 0.001$ ***	n.s.	n.s.	n.s.	n.s.	**	n.s.	*	*	*	***	n.s.	n.s.	*	*

Figure 3e+f **Redox**

Independent Cell	Sample	OxD_{roGFP1} [%]	E_{roGFP1} [mV]
1	NRF2 WT 2	95.07	-253.00
2	NRF2 WT 2	63.94	-283.64
3	NRF2 WT 2	35.45	-298.70
4	NRF2 WT 2	66.64	-282.11
5	NRF2 WT 2	53.13	-289.39
6	NRF2 WT 2	86.00	-267.68
7	NRF2 WT 2	43.64	-294.28
8	NRF2 WT 2	44.12	-294.03
9	NRF2 WT 2	50.41	-290.79
10	NRF2 WT 2	21.60	-307.55
11	NRF2 WT 2	21.32	-307.77
12	NRF2 WT 2	96.06	-249.98
13	NRF2 WT 2	46.85	-292.62
14	NRF2 WT 2	30.52	-301.57
15	NRF2 WT 2	27.59	-303.39
16	NRF2 WT 2	63.93	-283.65
1	NRF2 p.T80K	38.47	-297.03
2	NRF2 p.T80K	24.35	-305.56
3	NRF2 p.T80K	12.67	-315.79
4	NRF2 p.T80K	14.29	-314.01
5	NRF2 p.T80K	20.73	-308.22
6	NRF2 p.T80K	21.44	-307.67
7	NRF2 p.T80K	1412	-314.19
8	NRF2 p.T80K	21.97	-307.27
9	NRF2 p.T80K	6.14	-326.02
10	NRF2 p.T80K	22.15	-307.14
11	NRF2 p.T80K	20.78	-308.19
12	NRF2 p.T80K	43.35	-294.43
13	NRF2 p.T80K	12.46	-316.04
14	NRF2 p.T80K	11.47	-317.24
15	NRF2 p.T80K	24.99	-305.11
Mean	NRF2 WT 2	52.89	-287.51
	NRF2 p.T80K	20.63	-309.59
±SEM	NRF2 WT 2	6.05	4.36
	NRF2 p.T80K	2.56	2.06
**unpaired Welchs's t test (doesn't assume equal variances)**	$p \le 0.05$ considered significant	$T(20.162)=4.908$	$T(21.1304)=4.577$
	NRF2 p.T80K vs. WT 2	$p=0.000$	$p=0.000$
	$p \le 0.05$ *, $p \le 0.01$ **, $p \le 0.001$ ***	***	***

Supplementary Table 5a Statistic analysis details

Figure 4<br>Independent Experiment	Western blot<br>Sample	NRF2 [%]
1	DMSO	100.00
	Luteolin 50µM	4.31
2	DMSO	100.00
	Luteolin 50µM	8.66
3	DMSO	100.00
	Luteolin 50µM	6.61
Mean	DMSO	100.00
	Luteolin 50µM	6.53
±SEM	DMSO	0.00
	Luteolin 50µM	1.02
**unpaired Welchs's t test**	$p \le 0.05$ considered significant	$T(74.396)=4.487$
**(doesn't assume equal**	Luteolin 50µM vs. DMSO	$p=0.000$
**variances)**	$p \le 0.05$ *, $p \le 0.01$ **, $p \le 0.001$ ***	***

SFigure 4<br>Independent Experiment	Western blot<br>Sample	NRF2 [%]	KEAP1 [%]	G6PD [%]	AKR1B10 [%]	AKR1C1 [%]
1	NRF2 WT 1	100.00	100.00	100.00	100.00	100.00
	NRF2 WT 2	141.85	106.22	113.95	81.78	381.20
	NRF2 WT 3	112.36	42.95	204.49	63.83	174.89
	NRF2 WT 4	101.79	60.90	528.33	57.40	118.01
	NRF2 WT 5	76.84	80.02	708.56	47.06	271.55
	NRF2 p.T80K	259.03	135.76	3298.15	302.91	4540.47
2	NRF2 WT 1	100.00	100.00	100.00	100.00	100.00
	NRF2 WT 2	38.56	204.98	259.73	99.06	736.15
	NRF2 WT 3	91.98	78.02	773.47	58.67	225.35
	NRF2 WT 4	110.04	129.51	674.31	138.86	116.49
	NRF2 WT 5	91.14	69.35	486.57	100.48	139.16
	NRF2 p.T80K	133.95	157.34	2265.17	335.49	3778.56
3	NRF2 WT 1	100.00	100.00	100.00	100.00	100.00
	NRF2 WT 2	73.97	280.47	81.67	92.19	203.62
	NRF2 WT 3	78.03	286.05	136.44	63.85	279.00
	NRF2 WT 4	88.92	258.27	218.26	68.31	131.97
	NRF2 WT 5	52.39	245.02	135.48	58.51	310.39
	NRF2 p.T80K	221.72	504.76	1134.51	360.14	5612.26
Mean	NRF2 WT 1	100.00	100.00	100.00	100.00	100.00
	NRF2 WT 2	84.79	197.22	151.78	91.01	440.32
	NRF2 WT 3	94.12	135.67	371.46	62.11	226.41
	NRF2 WT 4	100.25	149.56	473.64	88.19	122.16
	NRF2 WT 5	73.45	131.46	443.54	68.68	240.37
	NRF2 p.T80K	204.90	265.95	2232.61	332.85	4643.76
±SEM	NRF2 WT 1	0.00	0.00	0.00	0.00	0.00
	NRF2 WT 2	30.30	50.45	54.77	5.02	156.54
	NRF2 WT 3	9.97	75.87	201.96	1.72	30.06
	NRF2 WT 4	6.15	57.85	134.46	25.53	4.93
	NRF2 WT 5	11.31	56.86	166.82	16.24	51.83
	NRF2 p.T80K	37.08	119.56	624.80	16.57	531.86
**One-Way Anova**	$p \le 0.05$ considered significant	$F(5,12)=5.359$, $p=0.008$	$F(5,12)=0.726$, $p=0.617$	$F(5,12)=8.009$, $p=0.002$	$F(5,12)=63.041$, $p=0.000$	$F(5,12)=52.648$, $p=0.000$
**multiple comparison post**	NRF2 p.T80K vs. WT 1	$p=0.034$	$p=0.556$	$p=0.002$	$p=0.000$	$p=0.000$
**hoc test**	NRF2 p.T80K vs. WT 2	$p=0.014$	$p=0.979$	$p=0.002$	$p=0.000$	$p=0.000$
	NRF2 p.T80K vs. WT 3	$p=0.024$	$p=0.769$	$p=0.006$	$p=0.000$	$p=0.000$
	NRF2 p.T80K vs. WT 4	$p=0.034$	$p=0.838$	$p=0.009$	$p=0.000$	$p=0.000$
	NRF2 p.T80K vs. WT 5	$p=0.007$	$p=0.746$	$p=0.008$	$p=0.000$	$p=0.000$
	NRF2 WT 1 vs. WT 2	$p=0.994$	$p=0.914$	$p=1.000$	$p=0.997$	$p=0.889$
	NRF2 WT 1 vs. WT 3	$p=1.000$	$p=0.999$	*0.981*	$p=0.457$	$p=0.998$
	NRF2 WT 1 vs. WT 4	$p=1.000$	$p=0.995$	*0.930*	$p=0.990$	$p=1.000$
	NRF2 WT 1 vs. WT 5	$p=0.937$	$p=0.999$	*0.949*	$p=0.640$	$p=0.997$
	$p \le 0.05$ *, $p \le 0.01$ **, $p \le 0.001$ ***	*	n.s.	**	***	***

SFigure 5<br>Independent Experiment	Western blot<br>Sample	NRF2 [%]
1	-	100.00
	Ascorbic acid 0.1 mM	47.96
	Ascorbic acid 0.25 mM	60.90
	Ascorbic acid 0.5 mM	104.92
	Ascorbic acid 0.75 mM	88.01
	Ascorbic acid 1.0 mM	85.16
2	-	100.00
	Ascorbic acid 0.1 mM	87.59
	Ascorbic acid 0.25 mM	64.55
	Ascorbic acid 0.5 mM	94.83
	Ascorbic acid 0.75 mM	61.82
	Ascorbic acid 1.0 mM	28.87
3	-	100.00
	Ascorbic acid 0.1 mM	129.08
	Ascorbic acid 0.25 mM	72.10
	Ascorbic acid 0.5 mM	95.82
	Ascorbic acid 0.75 mM	155.61
	Ascorbic acid 1.0 mM	139.46
Mean	-	100.00
	Ascorbic acid 0.1 mM	88.21
	Ascorbic acid 0.25 mM	65.85
	Ascorbic acid 0.5 mM	98.52
	Ascorbic acid 0.75 mM	101.81
	Ascorbic acid 1.0 mM	84.50
±SEM	-	0.00
	Ascorbic acid 0.1 mM	19.12
	Ascorbic acid 0.25 mM	2.69
	Ascorbic acid 0.5 mM	2.62
	Ascorbic acid 0.75 mM	22.81
	Ascorbic acid 1.0 mM	26.07
**One-Way Anova**	$p \le 0.05$ considered significant	$F(5,12)=0.470$, $p=0.791$
**multiple comparison post**	- vs. 0.1 mM Ascorbic acid	$p=0.998$
**hoc test**	- vs. 0.25 mM Ascorbic acid	$p=0.822$
	- vs. 0.5 mM Ascorbic acid	$p=1.000$
	- vs. 0.75 mM Ascorbic acid	$p=1.000$
	- vs. 1.0 mM Ascorbic acid	$p=0.992$
	$p \le 0.05$ *, $p \le 0.01$ **, $p \le 0.001$ ***	n.s.

Supplementary Table 5b Statistic analysis details

a Primer for Sanger sequencing validation

Gene	forward primer 5'-3'	reverse primer 5'-3'	amplicon [bp]	Target
NFE2L2	CTTGCCACACACAGTAACGC	CAGTCAGCGACGGAAAGAGT	538	DNA
NFE2L2	CCCAGCAGGACATGGATTTG	TGGGCAACCTGGGAGTAGTT	283	cDNA

b Primer for qRT-PCR

Gene	forward primer 5' - 3'	reverse primer 5' - 3'	amplicon [bp]
ACTB	TGACCCAGATCATGTTTGAG	ATCACGATGCCAGTGGTA	103
GAPDH	GTATGACAACAGCCTCAAGAT	GTCCTTCCACGATACCAAAG	104
PGK1	CTAACAAGCTGACGCTGGA	GACAGCAGCCTTAATCCTCTG	122
NFE2L2	ATCATGATGGACTTGGAGCTG	GCTCATACTCTTTCCGTCGC	145
KEAP1	CCAACTTCGCTGAGCAGATT	GCTGATGAGGGTCACCAGTT	137
GCLM	CTGTGTGATGCCACCAGATT	GCTTCTTGGAAACTTGCTTCA	108
GSR	ACAAGCTGGGTGGCACTT	ACCCTCACAACTTGGAAAGC	122
G6PD	AGAGCTTTTCCAGGGCGAT	CACCAGATGGTGGGGTAGAT	108
ME1	ACGAATTCATGGAGGCAGTT	GGAGACGAAATGCATTCACA	90
PRDX1	GCTGTTATGCCAGATGGTCAG	GGGCACACAAAGGTGAAGTC	104
TXNRD1	CAGCATGTCATGTGAGGACG	TTGAAGTCTGCCCTCCTGAT	148
ABCC1	TCATGCTCACTTTCTGGCTG	AATCAACCCTGTGATCCACC	149
HMOX1	GCCAGCAACAAAGTGCAAG	GAGTGTAAGGACCCATCGGA	105
AKR1B10	TTCTCGATCTGGAAGTGGCT	GGAAAAGCAACGTTCTTGGA	107
AKR1C1	TTGACTTGCAGAAATCCAGC	AAGCCAGGGCTCAAGTACAA	91

Supplementary Table 6 Primer sequence information. Detailed primer information for (**a**) Sanger Sequencing and (**b**) quantitative real time PCR. Oligonucleotides were designed for human material and purchased from Integrated DNA Technology (Leuven, Belgium).
